# Community Pharmacists’ Views on Their Roles in Mental Health Screening and Management in Malaysia

**DOI:** 10.1007/s10597-024-01337-9

**Published:** 2024-08-12

**Authors:** Shien Loong Mok, Jing Ying Chuah, Kun Jin Lee, Yee Dom Lim, Jamuna Rani Appalasamy, Pui San Saw, Amutha Selvaraj

**Affiliations:** School of Pharmacy, Monash University Malaysia, Building 2, Level 5 Jalan Lagoon Selatan, Bandar Sunway, Selangor 47500 Malaysia

**Keywords:** Pharmacist, Mental health, Screening, Management, Community pharmacy

## Abstract

Community pharmacists (CPs) are vital as primary healthcare providers, particularly in the screening and management of mental health issues. This study aimed to explore CPs’ views on mental health support for patients and the potential challenges in delivering mental health services. Malaysian CPs were recruited through purposive and snowballing sampling. Semi-structured interviews were recorded and transcribed verbatim. Data was thematically analyzed using NVivo 12 management software. Twenty CPs from Peninsular Malaysia were interviewed. Participants emphasized the importance of high-quality resources, comprehensive training and standardized tools to effectively provide mental healthcare services. Challenges identified were lack of knowledge and skills, absence of screening tools and social stigma and conservatism, particularly among older individuals. This study underscores the willingness of CPs taking a primary role in mental health services. However, collaboration with relevant stakeholders is crucial, aligning with national strategic plans for the program to be successful.

## Introduction

According to the Malaysian National Health and Morbidity Survey (NHMS) in 2015, the prevalence of mental health problems (i.e. depression and anxiety) recorded a significant two-fold rise from 10.6 to 29.2% over the past 10 years among those aged 16 years old and above (Press statement by Minister of Health Malaysia: Mental Health Problems in Malaysia, 2016). As primary healthcare providers, community pharmacists (CPs) play an important role in screening and managing mental health issues in the community. CPs can engage patients and actively support them to enhance their coping skills and mental well-being. Therefore, it is vital to evaluate the perspective of CPs regarding mental health provision and the barriers they encountered to strengthen their roles in helping patients with mental health issues.

In 2020, a survey among 96 CPs in Malaysia revealed that as high as 80% of the CPs agreed that mental illnesses should be perceived as something normal and nothing to be ashamed of. They expressed a neutral view towards patients with mental health problems and their willingness and confidence in managing mental health issues (Wong et al., 2020). Nevertheless, most of them emphasized the need for further training and education on mental health, such as counseling and pharmaceutical care related to psychotropic medications to facilitate mental health care and management in the community (Wong et al., 2020). Pharmacists can assist in opportunistic screening for mental health in the community setting, however the knowledge gaps need to be addressed via training and skills development (Rubio-Valera et al., 2014).

Apart from training requirements, appropriate counseling areas should also be designated in the mental health service provision by CPs. Private consultation rooms are essential for effective mental health counseling to allow active discussion. Previous studies have highlighted significant challenges in this regard. A study from the United States reports that most of the community pharmacies cannot prepare private consultation rooms due to space constraints (Calogero & Caley, 2017). In view of these shortcomings, some members of the public are less confident about the management of mental health issues by CPs (Calogero & Caley, 2017). Furthermore, there is a certain level of discrimination and stigmatization toward patients with mental disorders among some health professionals (Hanafiah & Van Bortel, 2015). Despite these findings, there is a notable gap in the literature regarding qualitative studies exploring the perceptions and experiences of health professionals and patients on the role of pharmacists in mental health (Fernandes et al., 2021). This study aims to address this gap by providing in-depth examination of these perspectives, thereby contributing to a more comprehensive understanding of the barriers and facilitators to effective mental health service provision by CPs. This study will offer valuable insights that could inform policy and practice, ultimately enhancing the capacity of CP to support mental health care. This study is particularly important as it seeks to bridge the existing knowledge gap and provide actionable recommendations to improve mental health services in the CP settings.

## Methodology

This qualitative study was context-dependent and took into account the viewpoints and values of key informants; who are the CPs. Based on the comprehensive literature reviews performed, a list of questions were established to address specific gaps and explore areas beyond the scope of existing surveys. Next, a semi-structured interview was performed among the participants. A phenomenological approach was used to explore details from the participant’s point of view (Fereday & Muir-Cochrane, 2006). Ethics approval was obtained from the Monash University Human Research Ethics Committee, with the project ID number 32,318.

The participants were selected via purposive sampling to recruit CPs with various demographic backgrounds. CPs who were fully registered pharmacists, owned and/or worked in community pharmacies, able to comprehend the interview session in English, and provided consent for the interview were deemed eligible. The recruitment was conducted by sending emails to personal contacts of the study team members as well as via the snowballing technique by sharing the invitation in various group chats with pharmacists. CPs who were interested were provided with an Explanatory Statement before they provided written consent to participate in this study. Before the interviews, the participants filled in relevant data about their demographic background details.

A semi-structured interview guide (Table 1) was developed after a literature review of the potential knowledge gaps of the topic in the current evidence pool. Two pilot- online interviews were conducted with community pharmacists who met the inclusion criteria to ensure content validity of the interview guide and techniques; however, these interviews were not included in the data collection and analysis. The data collected in the two pilot interviews were not included as part of the result. Based on the findings from the pilot interviews, the interview guide was amended accordingly. A definition of mental health was included in the guide, i.e. “*Mental health includes our emotional*,* psychological*,* and social well-being. It affects how we think*,* feel*,* and act. It also helps determine how we handle stress*,* relate to others*,* and make healthy choices*” (Centers for Disease Control and Prevention, 2024).

All interviews were conducted online; via zoom meeting. The interviews were audio recorded and transcribed verbatim. One researcher (MSL) conducted the interviews, whereas other researchers (CJY, LYD and LKJ) checked for accuracy and helped with the technical setting. Transcripts were reviewed to identify common underlying ideas and viewpoints that emerged from the quotes of each participant. Data was managed using NVivo 12 software and summarized into understandable codes and organized as tree nodes under pertinent topics, from which subthemes were derived. From these, subthemes were derived through a collaborative process among all authors to ensure agreement and consistency in the identified themes and subthemes that captured the underlying meaning of the data. When no new or emergent theme materials could be found in the transcripts, data saturation was deemed achieved.

**Table 1:** Interview guide

1. Generally, what is the interpretation of mental health? (Then, provide our mental health definition) What do you know about mental health? Stress? a. What is your perception or understanding of mental health issues in the community? b. What is the definition of mental health?
2. How do you identify that someone needs help and what is your response to them? a. Tell me more about common symptoms associated with mental health in the community setting. b. What preliminary method would you use to counsel patients (any specific tool)? i. What tools, guidelines, or protocols do you normally use to identify the patient’s mental health status? ii. Are you aware of certain screening tools? iii. Do you think you have sufficient knowledge to identify the severity of mental health problems? c. How frequently do you normally counsel someone who seeks your advice on mental health in the community pharmacy? d. How do you perceive the customer as having mental health issues?
3. What do you think about the need to provide mental health care services in community pharmacies?
4. What mental health care services are available in community pharmacies? a. What services have you offered to your customers? i. If yes, what protocols/services/ multidisciplinary approach (referral to GP, engagement with MOH/clinic)? (Note: proceed to question 5b to explore the importance) ii. If not, why? (Note: proceed to question 5 to explore potential barriers) b. Why do you think these services are important? c. Any non-pharmaceutical services or interventions provided for mental health? d. Provide some counseling points. Do they consult about adherence services?
5. What do you think are the common barriers/challenges/stigmas that community pharmacists face in the provision of mental health? (medical access, education, lack of knowledge, lack of resources and guidelines, etc.) a. What training or certification do you think community pharmacists should be equipped to deliver this service? i. Have you recently attended any training related to mental health care?
6. How do you think these barriers/challenges can be overcome? a. How would you overcome this situation (the common barriers mentioned by the pharmacist)? b. Do you see any involvement of the ministry/professional organizations to overcome this challenge? c. What is currently lacking in Malaysia for CPs in the provision of mental health care services? (skills enhancement)
7. If Malaysia needs your contribution as a pharmacist in mental health screening and management, will you be willing and available to participate in the role to provide mental health services to the community?

## Results

Overall, 20 interviews were conducted, each lasting approximately one hour, over a span of three months. The CPs were recruited from Pahang, Selangor, Penang, Malacca, and Wilayah Persekutuan Kuala Lumpur. The majority of the participants were females (60%) and between 25 and 35 years old (75%). The interviewees consisted of 95% Chinese and 5% Malay. As high as 60% of them worked in housing areas while the remaining worked in cities. Three-quarters (75%) of them worked in independent pharmacies while the rest were from chain pharmacies. Their work experience ranged from two to 18 years.

Six main themes emerged from the interviews, including: (1) CPs’ perspectives of understanding of mental health, (2) CPs as primary mental healthcare providers, (3) Challenges faced by CPs in provision of mental health services,, (4) CPs’ perspective on the social stigma of mental health, (5) CPs’ perspective on cost of service provision, and, (6) CPs’ availability and willingness to be involved in mental health service. The participants’ quotes were inserted in the following section as evidence to support each theme. (Refer Appendix for additional quotes). The following abbreviation was used, i.e. P1 = Participant 1, P2 = Participant 2, P3 = Participant 3, etc.

### Theme 1: CPs’ Perspectives of Understanding of Mental Health

The respondents expressed their understanding and shared their perspectives on mental health. Five participants mentioned that mental health is related to general coping skills in life.

…It is related to the person’s coping mechanism to events in his life and how he interprets the events. (P8)

Malaysians tend to live in a new environment, a new way of living, a new way of adapting to their life. So, some people might not be able to cope with the change or the changes… (P16).

Another nine participants related mental health to emotional aspects of feelings and well-being.

Mental health can connect to a positive mindset such as an optimistic mindset that is able to handle things rationally and positively or not to just take everything at a very pessimistic level. (P15)

Mental health is basically about our emotional qualities, maybe it varies among individuals, and everyone has different levels of mental health. (P19)

However, six participants perceived mental health as the presence of mental health disorders.

…Like those, especially anxiety or depression. Basically, just anxiety and depression in my definition. (P2)

Mental health is something that measures mental disorders, something like depression, anxiety, schizophrenia and central problems. (P3)

Next, most of the participants reported that they identified someone who was seeking help for mental health issues by observing their body language, gestures, and behaviors. Some of the observations included being aggressive, shouting, behaving in a rushing manner, and breathing rapidly.

They are very aggressive… (P11).

They are breathing very fast, talking very fast and then they seem very panicky. (P17)

However, some patients might also seek help directly by mentioning their issues and asking for advice regarding their mental health, such as ways to help them relax or calm down.

They are asking for certain advice according to their mental health. (P10)

Some customers are coming to the pharmacy, and they requested something for relaxation or something to calm them down… (P9).

On a more serious note, some participants mentioned that they had encountered suicidal patients who expressed their intention to commit suicide.

They have intention to commit suicide. (P12)

The frequency of CPs identifying mental health issues in the community was rather low based on the interview. Several CPs claimed that they rarely or never encountered any patients with mental health issues in their community pharmacies.

Yes. It’s very rare. So far, I have only met one that needs me to counsel on that. Then the other one was just, she mentioned it as part of her medical history. (P17)

### Theme 2: Community Pharmacists as Primary Mental Healthcare Providers

The majority of the participants agreed that CPs can play an important role in the provision of mental healthcare services in the community. Members of the public would prefer to visit community pharmacies before visiting general practitioners or specialists because of the convenience in terms of accessibility and shorter waiting time compared to clinics or hospitals. Moreover, CPs are placed in a more visible position that enables them to tackle mental health problems earlier.

…community pharmacies are the easiest accessible healthcare centers or health hub centers for the community. (P10)

“The community pharmacist also became more visible and presentable to more people in the community either in the rural or on the streets. (P10)”.

Nowadays, people always go to the pharmacy first before going to see a doctor, probably because we get to spend more time with them and we ask them more questions. (P1)

If they feel like having health issues they will not go to see the doctor straight, they will go to a pharmacy first and it is more accessible. I guess it is also free and makes it easier for them. (P17)

Currently, the majority of CPs offer mental health services using their approach without any diagnostic tools.

So far, I haven’t got any tools to diagnose patients. So, just according to my experience. (P18)

During the counseling, if the CPs felt that any cases need to be referred, they would inform the patients directly and issue a referral letter for them to see a specialist.

If we feel that the case is chronic or severe, we will write a referral letter to the patient to refer them to the psychiatrist. (P13)

### Theme 3: Challenges Faced by Community Pharmacists in Provision of Mental Health Services

Most of the CPs expressed a lack of time for them to focus on the customers with mental health issues due to a heavy workload in the daily operation of community pharmacies.

We don’t have enough time for every customer. Sometimes the customers want to talk more but we have no sufficient time to entertain them because we are on a first come first serve basis. (P12)

Generally, almost all participants reported that no proper training on mental health was available to them through pharmacy organizations, societies and stakeholders. They felt that they needed to be well-trained before offering such services in their pharmacies but they were unsure of where or who to approach for this purpose.

We do not know how to approach it. We do not know what to start and where to start.(P10).

Perhaps having community pharmacy organizations to organize like mental health workshops, or mental health training for community pharmacies…(P3).

Other than the opportunity to attend more training, the majority of participants expressed the wish for the government or professional institutions to establish a standardized guideline on how to screen patients with mental health issues in the community. Such guidelines will enable healthcare professionals such as doctors and CPs to provide better care for patients.

Government should come up with a guideline and a health screening to include mental health screening as part of the overall health screening or a guideline for community pharmacies, clinics and hospitals to follow so that we can actually identify red flag symptoms and to do a proper screening… (P4).

### Theme 4: CP’s Perspective on the Social Stigma of Mental Health

With regard to social stigma, the participants shared that most of the older people refused to talk about mental health or be clinically diagnosed. They feel mental health is taboo and a sensitive issue, thus, many of them refrained from sharing their mental health issues with CPs. In contrast, the younger generation is more willing to acknowledge mental health issues and take action to manage them.

Stigmas are definitely. The younger generation is actually more open but not for the older generation. They think mental health is a special issue. (P15).

Apart from that, most of the CPs claimed that Malaysians are conservative, especially with regard to mental health discussions. They tend to ignore questions that may elicit social stigmas during the conversation.

Mental health is still known in Malaysia as a very complex taboo topic, like a very sensitive topic…. It takes time to build the rapport until patients are willing to open up to you. (P3)

I think Malaysians for us in general it’s still not normal to admit that actually we need help. (P4)

### Theme 5: CP’s Perspective on the Cost of Service Provision

With regards to the cost of services provided by CPs, there is also a lack of outcomes such as direct reimbursement for CPs to provide mental health services. For example, CPs do not receive extra payment when giving detailed consultations to patients with mental health issues. Some CPs viewed this as the main reason why CPs are less keen to pay extra effort in the field of mental health.

…The pharmacists did not get extra pay for such counseling, so most of the pharmacists did not put much effort. (P8)

As above mentioned, CPs’ knowledge of mental health screening and management can be largely improved by training courses, especially those customized for community providers such as CPs. However, most of these courses are expensive and not always affordable. Therefore, most of them never attended any mental health-related courses.

…this course not a lot of us are joining because for younger pharmacists who may want to join it is quite expensive and it is not cheap for them. (P6)

### Theme 6: CPs’ Availability and Willingness to be Involved in Mental Health Service

On a positive note, all participants expressed their willingness to be involved in the provision of mental health services to the community. However, as stated by two participants, certain criteria such as knowledge enhancement and protected time should be fulfilled to ensure a smooth provision of mental health services.

If there is such a role, I think it will be good to participate. (P5)

I am willing to help but first we need to improve ourselves, like how to screen, how to help, how to refer and when to refer. (P11)

If the company allows me to have more time on mental health service, of course I can, because this is part of my career. (P20)

## Discussion

**Fig. 1:**
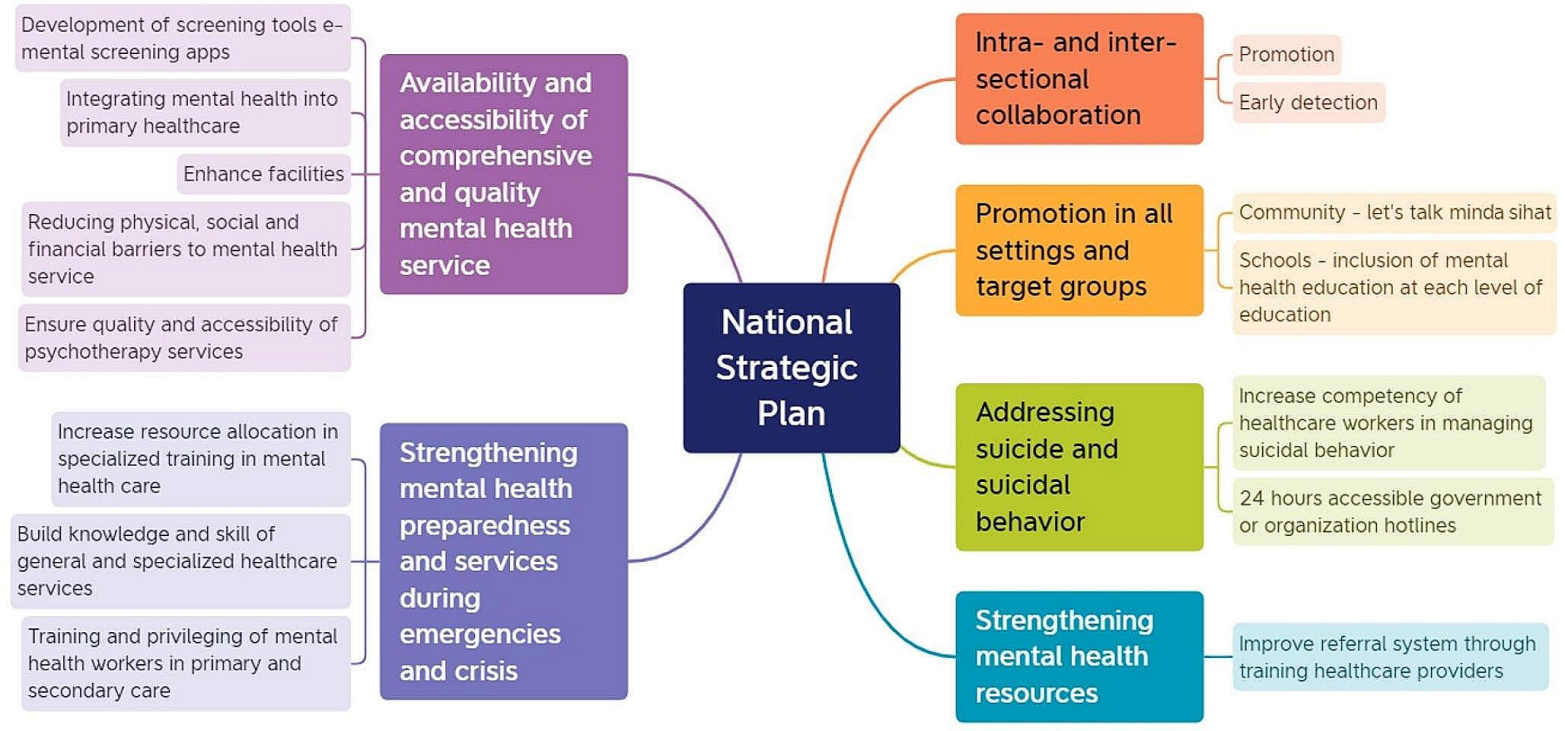
Mapping of themes to the national strategic plan for mental health 2020–2025 (6)

In this study, the majority of the CPs mentioned that they detected mental health issues among the patients based on body language, gestures, and behaviors. Due to the lack of proper screening tools, most of the CPs determined the severity of mental health issues among the patients based on observation. They would recommend a referral to specialists when deemed necessary (Wong et al., 2020). In other words, they evaluated the patient’s condition using their approach and experience. These situations concurred with the recommendation of the National Strategic Plan for Mental Health (Fig. 1) in which a standardized screening tool should be established to facilitate community healthcare providers such as CPs to screen and assess the severity of mental health issues among walk-in patients (Malaysia MoH, 2020). By doing this, CPs will be able to assist patients for referral to the required healthcare facilities provided by government or private sectors. As the first point of contact for the community whenever they have a concern, CPs represent a more convenient and accessible option for the public. Furthermore, patients may find the services offered by CPs more affordable (Hassell et al., 1997; Rubio-Valera et al., 2014). In addition, it is more time-saving as patients can just walk into the pharmacy without any appointments (Rubio-Valera et al., 2014). As a result, community pharmacy represents an effective screening station for early detection of mental health issues (Rubio-Valera et al., 2014).

However, CPs mentioned the challenges in terms of insufficient time and attention because mental health-related services often require detailed counseling with patients (Wong et al., 2020). The operation in community pharmacies is often fast-paced, thus making it challenging for CPs to dedicate sufficient time to patients with mental health disorders. To address this barrier, it is vital to enhance the referral system to enable CPs to efficiently collaborate with mental health specialists (Malaysia MoH, 2020). This improvement will allow CPs to provide initial support and screening for mental health issues and then refer patients who need comprehensive counseling services to relevant specialists, thereby ensuring a more integrated approach to mental healthcare. CPs can utilize standardized assessment tools and training to better identify patients requiring extensive counseling.

In the local setting, mental illness has not been well understood by society historically, resulting in social stigmas against patients with mental health disorders. Malaysian society is generally considered conservative with regard to mental health issues (Osman, 2017). This is especially true for the elderly population who are more reluctant to accept mental health diseases as a type of illness. All these social stigmatization issues are compounded by a lack of awareness of mental health issues in the community. Similar trends are observed globally, with studies indicating that mental health stigma, particularly among the older population, is a widespread issue affecting various countries. This global perspective highlights the universal challenge of addressing mental health stigma and the need for increased awareness and education efforts worldwide (Conner et al., 2010, Stewart et al., 2015, Park et al., 2015). As a result, CPs should play a role to initiate stigma reduction initiatives in the community so that society is more comfortable and willing to speak out about mental health concerns. This is in line with the National Strategic Plan that recommended the promotion of mental health in all settings, such as organizing “Let’s Talk Minda Sihat’’ campaigns and incorporating mental health education in all stages of education (6). This was also supported by our findings in which most CPs claimed to have only been exposed to a minimum level of mental health topics during their undergraduate years. Thus, community pharmacists in Malaysia will require new education exposure and training skills such as mental health first aid (MHFA) to take on significant roles in mental health care as practiced by developing countries such as Canada and Australia (El Den S. et.al 2021).

Besides that, two aspects of financial barriers were highlighted in the study results. From the patient’s perspective, the treatment cost of psychiatrists can be a financial burden, thus hindering many patients to seek help (Raaj et al., 2021). This financial concern can also affect their willingness to engage with a CP about their mental health, as they might fear being referred to a costly specialist. Therefore, accessible and affordable mental health services delivered by CPs can play a crucial role in initial support and ongoing care. Some patients also found the recommended supplements from CPs to be expensive such as omega-3 EPA + DHA, phytosterols, calcium and Vitamin D, though Malaysia is yet to overcome its malnutrition burden and positive growth of dietary supplements (Weerasena et al., 2021). Nevertheless, the efficacy of these supplements for mental health treatment and management are yet to be explored. Our study participants indicated that these would increase financial barriers and additionally affect a patient’s adherence, and subsequently the outcome of the treatment. The CPs highlighted the low level of reward when compared to the efforts required when managing mental health-related cases. Since there are no direct reimbursement or financial incentives for mental health patient counseling which can be time and effort-consuming, CPs might be more inclined to spend time servicing other customers to obtain more financial profits.

Furthermore, mental health training courses are usually required to be paid at CPs own expense. However, if there are free courses, they are keen to attend these training sessions for the advancement of knowledge and skills in managing mental health cases. The national strategic guidelines mooted the idea of training and privileging healthcare workers in primary and secondary healthcare service facilities to help in tackling mental health conditions (Malaysia MoH, 2020). Despite the existing barriers, all participants expressed a keen interest in providing mental healthcare services in community settings if they have access to proper guidelines and training, a mental health-friendly environment, and financial support throughout the process.

This study has several limitations. Firstly, we acknowledge that the challenges observed could potentially be influenced by the geographic locations of the community pharmacies where the participating CPs practiced. Secondly, the perspectives shared by the CPs were based on their personal experiences with mental health services. Furthermore, due to the purposive recruitment of the participants, there is a possibility that the viewpoints expressed could be skewed. However, we believe despite the limitations, the findings of the study can be used to provide information on reformation of practices and policies pertaining to mental health services in community settings. Future studies could look into the education and training needs relevant for CPs to provide initial support and screening for mental health issues.

## Conclusion

The study provided useful insights into the current scenario of the screening and management of mental health problems in community pharmacies. All the participants in this study reported a readiness and willingness to be involved as a primary care provider of mental health services. However, various suggestions were made on necessary steps aligned with the national strategic plan that must be taken by the relevant stakeholders and policy holders such as the government agencies and healthcare providers to facilitate the provision of accessible and quality mental health care services in the community pharmacy setting.

## Appendix - Additional Participants’ Quotes

**Table:**

Theme	Quotation
Community pharmacist as primary mental healthcare providers	“…We are always like front liners. Nowadays, people always go to the pharmacy first before going to see a doctor, probably because we get to spend more time with them and we ask them more questions…” (P1)
	“…CPs are the first people to be able to notice or observe when someone goes from mentally healthy to downhill, especially for regular customers because we see them almost every day or so frequently in a week…” (P8)
	“…the community pharmacist also became more visible and presentable to more people in the community either in the rural or the streets…” (P10)
	“…Community pharmacists are frontliners to meet all those patients with health problems. So, when we meet these patients, actually, we can educate them. They might have this type of mental health problems and then they need to see a doctor. Most of the time they don’t know that they actually have this type of mental problem. So, we play an important role in educating them, in identifying the symptoms…” (P18)
Challenges faced by community pharmacists in provision of mental health services	“…Unfortunately, even in community pharmacies, or even hospitals and clinics, we don’t exactly have a proper screening tool to identify the science of this…” (P4)
	“…We don’t have proper training about that and the knowledge is just university level. Even in hospitals, the diagnosis part is normally done by a doctor, and we don’t even have the chance to counsel. Rarely…” (P3)
	“…We can know better and how to handle mental health problems like having a proper guideline that we can follow and provide proper counseling to them…” (P9)
CPs’ perspectives of understanding of mental health	“If they are having the mild type, you can hardly recognize whether they have this mental health issue unless it is a severe type…” (P15)
	“…Indirectly they are trying to portray their third person scenario by saying their friend has this and that…” (P10)
	“…From their nonverbal kind of communication or gestures, you will probably know that they are in a rush for time, or they are worrying about something at home…” (P16)
	“…We can ask more questions so that we can know what kind of problem they are having…” (P5)
CP’s perspective on social stigma on mental health	“…Mental health is still known in Malaysia as a very complex taboo topic, like a very sensitive topic. As a stranger it is a bit hard for them to open up their feelings. So I think it takes time to build the rapport until patients are willing to open up to you…” (P3)
	“…Mental health is a very conservative thing, a lot of Malaysians would not want to seek help even if they know they have the condition…” (P11)
	“…I think one of the main challenges from the patient is to share this kind of information. It’s actually the stigma…” (P16)
	“…They are less comfortable to speak up when there is a huge crowd unless there is a special counseling room…” (P8)
CP’s perspective on the cost of service provision	“…I think many refuse to visit a psychiatrist because of the cost as it is very expensive. And I have no idea about the market price or reasonable price to visit a psychiatrist as I only know a few of them. If they are low income, there are high chances to refuse to visit the psychiatrist…” (P7)
	“…that depends on whether the customer has any budget for some supplements or not, some don’t have a budget, and some are not cheap as well as herbal ingredients, it is more than RM50 and above…” (P14)
	“…The pharmacists did not get extra paid for such counseling, so most of the pharmacists did not put much effort…” (P8)
	“…Not a lot of us are joining these courses because for younger pharmacists who may want to join it is quite expensive and it is not cheap for them…” (P6)
CPs availability and willingness to be involved in mental health service	“Of course, yes.” (P17)
	“If the company can give me more time on these, of course I can. But then the problem is we have a heavy workload and we have limited time. So, if the company allows, of course I can, because this is part of my career.” (P20)
	“Yes, of course. Because this is our role as community pharmacies to provide help to the public. Of course we do. We are more than willing to help in this problem.” (P19)
	“If there is such a role, I think it will be good to participate.” (P5)
