# Correction: Community Pharmacists’ Views on Their Roles in Mental Health Screening and Management in Malaysia

**DOI:** 10.1007/s10597-024-01358-4

**Published:** 2024-09-11

**Authors:** Shien Loong Mok, Jing Ying Chuah, Kun Jin Lee, Yee Dom Lim, Jamuna Rani Appalasamy, Pui San Saw, Amutha Selvaraj

**Affiliations:** School of Pharmacy, Monash University Malaysia, Building 2, Level 5 Jalan Lagoon Selatan, Bandar Sunway, Subang Jaya, Selangor 47500 Malaysia

**Correction: Community Mental Health Journal**

10.1007/s10597-024-01337-9

The original version of this article unfortunately contained error in Fig. 1. The spelling of “minta sihat” should read as “minda sihat”.

The corrected figure is given here:

**Fig. 1:**
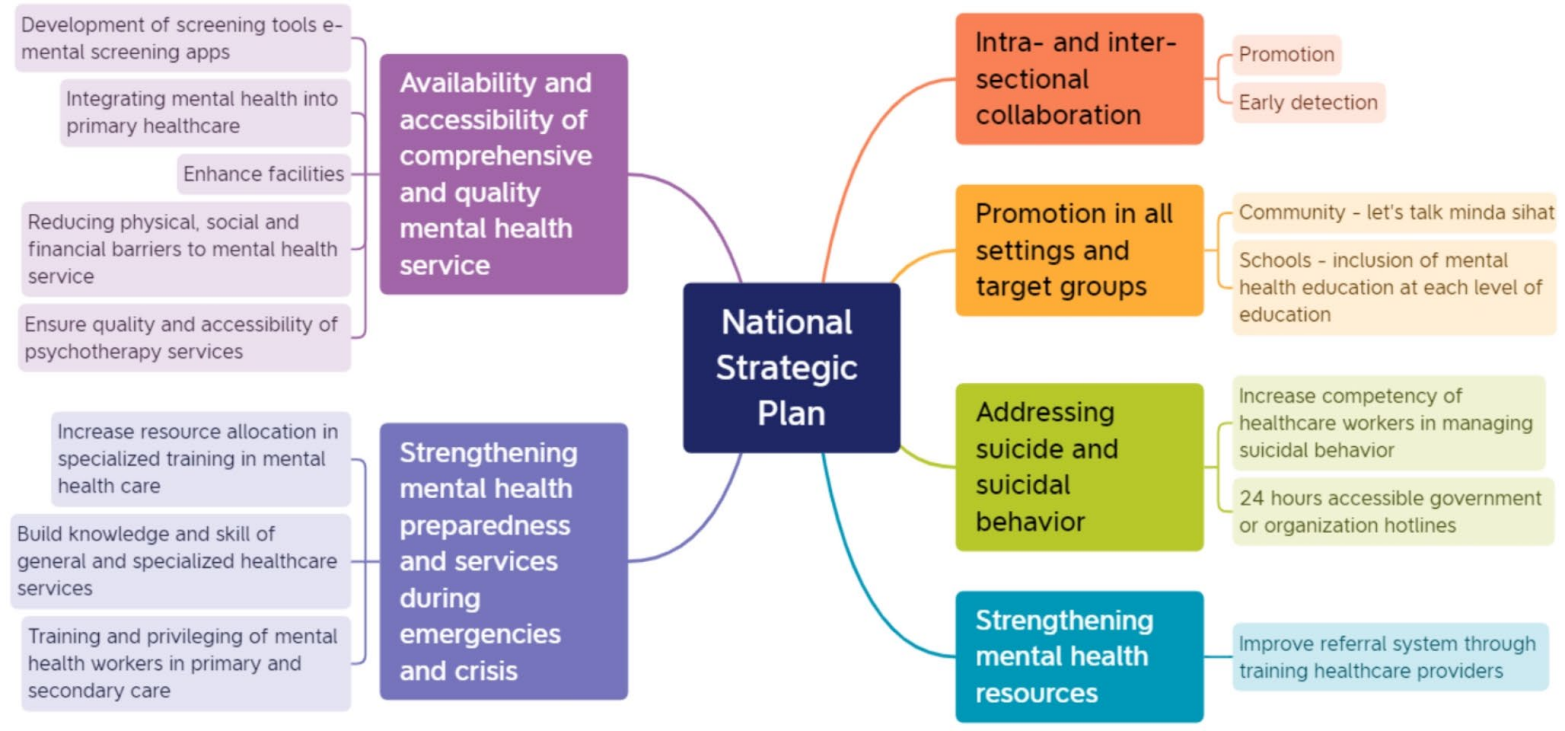
Mapping of themes to the national strategic plan for mental health 2020–2025 (6)

The original article has been corrected.

